# Ammonium 2-amino­pyrazine-3-carboxyl­ate

**DOI:** 10.1107/S1600536811010865

**Published:** 2011-03-26

**Authors:** Martin Lutz, Arjen J. Jakobi

**Affiliations:** a Bijvoet Center for Biomolecular Research, Crystal and Structural Chemistry, Faculty of Science, Utrecht University, Padualaan 8, 3584 CH Utrecht, The Netherlands

## Abstract

The title compound NH_4_^+^·C_5_H_4_N_3_O_2_^−^ crystallizes with two formula units in the asymmetric unit. In each anion, the carboxyl­ate is deprotonated and the planar amino group [angle sums of 359 (3) and 355 (3)° at N] remains protonated. In the crystal, the cations and anions are bridged by N—H⋯O and N—H⋯N hydrogen bonds, forming a three-dimensional network.

## Related literature

For the crystal structure of the free acid, see: Dobson & Gerkin (1996 ▶); Ptasiewicz-Bak & Leciejewicz (1997 ▶). For the metal complex with nickel, see: Ptasiewicz-Bak & Leciejewicz (1999 ▶). For the coordination chemistry of 2-pyrazine­carb­oxy­lic acid, see: Ptasiewicz-Bak *et al.* (1995 ▶); Ellsworth & zur Loye (2008 ▶). In the present study a half-normal probability plot (Abrahams & Keve, 1971 ▶), a quaternion fit (Mackay, 1984 ▶) and rigid-body analysis (Schomaker & Trueblood, 1998 ▶) have been used.
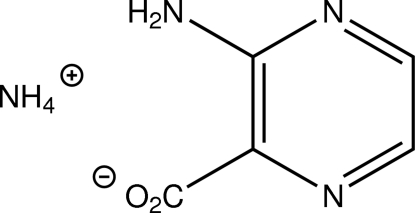


## Experimental

### 

#### Crystal data

NH_4_^+^·C_5_H_4_N_3_O_2_^−^
*M* *_r_* = 156.15
Orthorhombic, 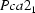

*a* = 12.5066 (6) Å
*b* = 3.8833 (2) Å
*c* = 27.9659 (14) Å
*V* = 1358.22 (12) Å^3^
*Z* = 8
Mo *K*α radiation
μ = 0.12 mm^−1^
*T* = 150 K
0.40 × 0.19 × 0.09 mm

#### Data collection

Bruker Kappa APEXII diffractometer
Absorption correction: multi-scan (*SADABS*; Sheldrick, 2008*a* ▶) *T*_min_ = 0.70, *T*_max_ = 0.75
16898 measured reflections
1580 independent reflections
1540 reflections with *I* > 2σ(*I*)
*R*_int_ = 0.018

#### Refinement

*R*[*F* ^2^ > 2σ(*F* ^2^)] = 0.026
*wR*(*F* ^2^) = 0.072
*S* = 1.05
1580 reflections
247 parameters
1 restraint
H atoms treated by a mixture of independent and constrained refinement
Δρ_max_ = 0.33 e Å^−3^
Δρ_min_ = −0.16 e Å^−3^

### 

Data collection: *APEX2* (Bruker, 2010 ▶); cell refinement: *SAINT* (Bruker, 2010 ▶); data reduction: *SAINT*; program(s) used to solve structure: *SHELXS97* (Sheldrick, 2008*b* ▶); program(s) used to refine structure: *SHELXL97* (Sheldrick, 2008*b* ▶); molecular graphics: *PLATON* (Spek, 2009 ▶) and *Mercury* (Macrae *et al.*, 2006 ▶); software used to prepare material for publication: manual editing of *SHELXL* cif file.

## Supplementary Material

Crystal structure: contains datablocks I, global. DOI: 10.1107/S1600536811010865/zl2357sup1.cif

Structure factors: contains datablocks I. DOI: 10.1107/S1600536811010865/zl2357Isup2.hkl

Additional supplementary materials:  crystallographic information; 3D view; checkCIF report

## Figures and Tables

**Table 1:** Hydrogen-bond geometry (Å, °)

*D*—H⋯*A*	*D*—H	H⋯*A*	*D*⋯*A*	*D*—H⋯*A*
N31—H31*A*⋯N22^i^	0.92 (3)	2.20 (3)	3.103 (2)	169 (2)
N31—H31*B*⋯O21	0.90 (3)	2.07 (3)	2.726 (2)	129 (2)
N32—H32*A*⋯N21^ii^	0.88 (3)	2.23 (3)	3.100 (2)	168 (2)
N32—H32*B*⋯O22	0.86 (3)	2.06 (3)	2.686 (2)	129 (2)
N3—H3*B*⋯O21	0.93 (3)	1.97 (3)	2.849 (2)	157 (2)
N3—H3*C*⋯O11^iii^	0.96 (3)	2.58 (3)	3.287 (2)	131 (2)
N3—H3*C*⋯N11^iii^	0.96 (3)	2.00 (3)	2.909 (2)	159 (2)
N3—H3*D*⋯O11^iv^	0.89 (3)	2.13 (3)	2.944 (2)	152 (2)
N4—H4*A*⋯O12	0.86 (3)	2.13 (3)	2.897 (2)	148 (3)
N4—H4*A*⋯N12	0.86 (3)	2.23 (3)	2.912 (2)	135 (3)
N4—H4*B*⋯O11	0.95 (4)	1.86 (4)	2.793 (2)	166 (3)
N4—H4*C*⋯O11^v^	0.84 (3)	2.01 (4)	2.839 (2)	170 (3)
N4—H4*D*⋯O22^vi^	0.87 (3)	1.87 (3)	2.742 (2)	176 (3)

Symmetry codes: (i) 
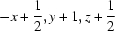
; (ii) 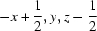; (iii) 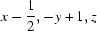; (iv) 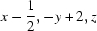; (v) 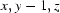; (vi) 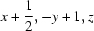.

**Fig. 1.**
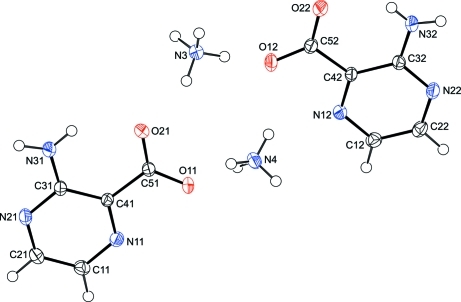
: Displacement ellipsoid plot of (I). View along the b axis. Non-hydrogen atoms are drawn at the 50% probability level; H atoms are drawn as spheres with arbitrary radii.

**Fig. 2.**
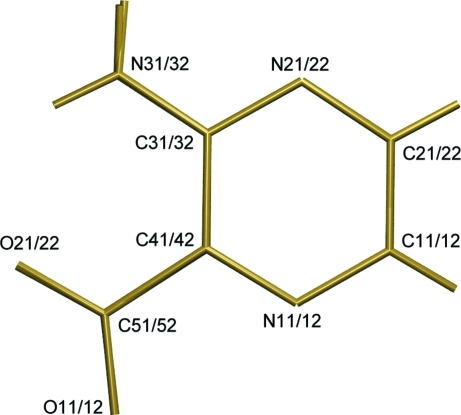
: Quaternion fit (Mackay, 1984) of the two independent anions in (I). One of the molecules is inverted. The r.m.s. deviation of the fit is 0.041 Å.

**Fig. 3.**
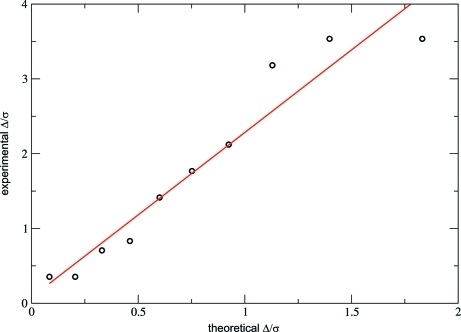
: Half-normal probability plot (Abrahams & Keve, 1971) of the bond lengths of the two independent molecules of (I). On the vertical axis are the experimental Δ/σ data, on the horizontal axis the theoretical expectation values. Linear regression results in a slope of 2.2 and an intercept of 0.08.

**Fig. 4.**
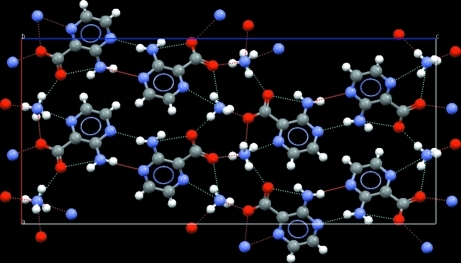
: Hydrogen bonding interactions in the crystal structure of (I). View along the b axis.

## supplementary crystallographic information

### Comment

2-Pyrazinecarboxylic acid is a complexation reagent in transition metal chemistry (Ptasiewicz-Bak *et al.*, 1995) with a large variety of coordination modes (Ellsworth & zur Loye, 2008). The corresponding 3-aminopyrazine-2-carboxylic has been used in a similar way for the complexation of nickel (Ptasiewicz-Bak & Leciejewicz, 1999). The crystal structure of the free acid has been determined by Dobson & Gerkin (1996) and Ptasiewicz-Bak & Leciejewicz (1997).

The asymmetric unit of the crystal structure of the title compound (I) consists of two formula units (*Z*' = 2). The anions are essentially planar with a maximal deviation from the least-squares plane of 0.059 (2) and 0.093 (1) Å for the two molecules, respectively (Fig. 1). The molecular planes form angles of 5.54 (3) and 0.58 (3)° with the *c* axis. Also the amino moieties are planar with angle sums of 359 (3) and 355 (3)° at N31 and N32.

The two independent molecules are very similar, as can be seen in a quaternion fit (Fig. 2). This allows the generation of a half-normal probability plot (Fig. 3). The largest differences between the two molecules are in the C–O distances (Δ = 3.5σ). A possible explanation is the different hydrogen bonding situation of the four O atoms. If the anions in (I) are compared with the neutral molecule of the free acid (Dobson & Gerkin, 1996) the geometries are again very similar. As expected, the only difference is in the carboxylate, which is deprotonated in (I) and protonated in the free acid. The distances C51–O11 and C52–O12 in (I) are 1.266 (2) and 1.256 (2)Å compared to the C–OH distance of 1.328 (2)Å in the free acid. This is accompanied by a change of the corresponding C–C–O angles, which are 116.01 (14) and 116.79 (14)° in (I) compared to 118.20 (10)° in the free acid.

The two independent molecules in (I) can be modelled by rigid body model using the program THMA11 (Schomaker & Trueblood, 1998). The fit of this TLS model is good, as indicated by *R*-values (*R*={[Σ(wΔU)^2^]/[Σ(wU_obs_)^2^]}^1/2^) of 0.080 and 0.085 for the two molecules. The two molecules can thus be appropriately described as rigid bodies. The T tensor has eigenvalues of 0.01925, 0.01329, and 0.01076 Å^2^ for the first independent molecule in (I), and 0.02060, 0.01368, and 0.01227 Å^2^ for the second molecule. The *L* tensor has eigenvalues of 13.62, 6.42, and 4.28 deg.^2^ for the first molecule, and 12.96, 7.20, and 4.22 deg.^2^ for the second molecule.

The amino moieties of the anions act as donors of two hydrogen bonds, respecively. One is intramolecular to the carboxylate [graph set S_1_^1^(6)], and one is intermolecular to a pyrazine N atom [graph set D_1_^1^(2)]. Overall, this results in one-dimensional hydrogen-bonded chains along the *b* axis. These chains are interconnected by the ammonium cations to form a three-dimensional network. Hydrogen atoms H3C and H4A of the ammonium cations are involved in bifurcated hydrogen bonds (Table 1, Fig. 4).

### Experimental

212 mg of 2-aminopyrazine-3-carboxylic acid were suspended in 20 ml water. A concentrated solution of ammonium hydroxide was added dropwise until the suspension became clear. Slow evaporation at room temperature gave crystals of (I) suitable for the diffraction experiment.

### Refinement

During the intensity integration, a small second crystal fragment has been ignored (less than 5% occupancy). Friedel pairs have been averaged prior to the refinement.

Hydrogen atoms were located in difference Fourier maps. N—H hydrogen atoms were refined freely with isotropic displacement parameters. C—H hydrogen atoms were refined using a riding model with C—H = 0.95 Å and with *U*_iso_(H) = 1.2 times *U*_eq_(C).

### Crystal data

**Table:**

NH_4_^+^·C_5_H_4_N_3_O_2_^−^	*F*(000) = 656
*M**_r_* = 156.15	*D*_x_ = 1.527 Mg m^−^^3^
Orthorhombic, *P**c**a*2_1_	Mo *K*α radiation, λ = 0.71073 Å
Hall symbol: P 2c -2ac	Cell parameters from 9416 reflections
*a* = 12.5066 (6) Å	θ = 2.9–27.5°
*b* = 3.8833 (2) Å	µ = 0.12 mm^−^^1^
*c* = 27.9659 (14) Å	*T* = 150 K
*V* = 1358.22 (12) Å^3^	Plate, colourless
*Z* = 8	0.40 × 0.19 × 0.09 mm

### Data collection

**Table:**

Bruker Kappa APEXII diffractometer	1580 independent reflections
Radiation source: fine-focus sealed tube	1540 reflections with *I* > 2σ(*I*)
graphite	*R*_int_ = 0.018
φ and ω scans	θ_max_ = 27.5°, θ_min_ = 2.9°
Absorption correction: multi-scan (*SADABS*; Sheldrick, 2008*a*)	*h* = −16→15
*T*_min_ = 0.70, *T*_max_ = 0.75	*k* = −4→5
16898 measured reflections	*l* = −36→36

### Refinement

**Table:**

Refinement on *F*^2^	Primary atom site location: structure-invariant direct methods
Least-squares matrix: full	Secondary atom site location: difference Fourier map
*R*[*F*^2^ > 2σ(*F*^2^)] = 0.026	Hydrogen site location: difference Fourier map
*wR*(*F*^2^) = 0.072	H atoms treated by a mixture of independent and constrained refinement
*S* = 1.05	*w* = 1/[σ^2^(*F*_o_^2^) + (0.0562*P*)^2^ + 0.1369*P*] where *P* = (*F*_o_^2^ + 2*F*_c_^2^)/3
1580 reflections	(Δ/σ)_max_ = 0.001
247 parameters	Δρ_max_ = 0.33 e Å^−^^3^
1 restraint	Δρ_min_ = −0.16 e Å^−^^3^

### Special details

**Table:**

Geometry. All e.s.d.'s (except the e.s.d. in the dihedral angle between two l.s. planes) are estimated using the full covariance matrix. The cell e.s.d.'s are taken into account individually in the estimation of e.s.d.'s in distances, angles and torsion angles; correlations between e.s.d.'s in cell parameters are only used when they are defined by crystal symmetry. An approximate (isotropic) treatment of cell e.s.d.'s is used for estimating e.s.d.'s involving l.s. planes.
Refinement. Refinement of *F*^2^ against ALL reflections. The weighted *R*-factor *wR* and goodness of fit *S* are based on *F*^2^, conventional *R*-factors *R* are based on *F*, with *F* set to zero for negative *F*^2^. The threshold expression of *F*^2^ > σ(*F*^2^) is used only for calculating *R*-factors(gt) *etc*. and is not relevant to the choice of reflections for refinement. *R*-factors based on *F*^2^ are statistically about twice as large as those based on *F*, and *R*- factors based on ALL data will be even larger.

### Fractional atomic coordinates and isotropic or equivalent isotropic displacement parameters (Å^2^)

**Table:**

	*x*	*y*	*z*	*U*_iso_*/*U*_eq_	
O11	0.42965 (10)	0.7629 (3)	0.54691 (4)	0.0224 (3)	
O21	0.30439 (11)	0.9858 (4)	0.59468 (5)	0.0238 (3)	
N11	0.55372 (12)	0.5954 (4)	0.62042 (5)	0.0188 (3)	
N21	0.50221 (12)	0.7550 (4)	0.71454 (5)	0.0200 (3)	
N31	0.34689 (13)	1.0186 (4)	0.69017 (5)	0.0224 (3)	
H31A	0.338 (2)	1.088 (7)	0.7212 (11)	0.031 (6)*	
H31B	0.304 (2)	1.100 (7)	0.6670 (10)	0.032 (7)*	
C11	0.61962 (15)	0.5125 (5)	0.65636 (6)	0.0211 (3)	
H11	0.6854	0.3996	0.6498	0.025*	
C21	0.59216 (14)	0.5908 (5)	0.70302 (6)	0.0209 (3)	
H21	0.6395	0.5246	0.7279	0.025*	
C31	0.43627 (13)	0.8473 (4)	0.67848 (6)	0.0164 (3)	
C41	0.46320 (13)	0.7604 (4)	0.63013 (6)	0.0156 (3)	
C51	0.39253 (13)	0.8432 (4)	0.58752 (6)	0.0174 (3)	
O12	0.14633 (10)	0.3689 (4)	0.46142 (4)	0.0245 (3)	
O22	0.02147 (11)	0.5588 (4)	0.41050 (5)	0.0281 (3)	
N12	0.26120 (11)	0.1003 (4)	0.39032 (5)	0.0188 (3)	
N22	0.21177 (12)	0.2151 (4)	0.29449 (5)	0.0207 (3)	
N32	0.05731 (12)	0.4997 (4)	0.31616 (6)	0.0232 (3)	
H32A	0.0512 (19)	0.574 (6)	0.2864 (10)	0.026 (6)*	
H32B	0.020 (2)	0.611 (7)	0.3371 (10)	0.035 (7)*	
C12	0.32517 (14)	−0.0150 (5)	0.35539 (7)	0.0211 (3)	
H12	0.3883	−0.1386	0.3632	0.025*	
C22	0.29979 (14)	0.0451 (5)	0.30788 (7)	0.0216 (4)	
H22	0.3469	−0.0379	0.2838	0.026*	
C32	0.14642 (13)	0.3310 (4)	0.32951 (6)	0.0175 (3)	
C42	0.17362 (13)	0.2711 (4)	0.37884 (5)	0.0163 (3)	
C52	0.10825 (13)	0.4099 (4)	0.42027 (6)	0.0189 (3)	
N3	0.12272 (12)	0.8079 (4)	0.53838 (5)	0.0201 (3)	
H3A	0.133 (2)	0.702 (7)	0.5094 (11)	0.032 (6)*	
H3B	0.189 (2)	0.888 (7)	0.5486 (10)	0.032 (6)*	
H3C	0.086 (2)	0.660 (7)	0.5602 (11)	0.037 (6)*	
H3D	0.079 (2)	0.986 (7)	0.5356 (9)	0.034 (7)*	
N4	0.37274 (14)	0.2716 (5)	0.47857 (6)	0.0249 (3)	
H4A	0.312 (3)	0.249 (7)	0.4640 (11)	0.043 (8)*	
H4B	0.380 (2)	0.443 (10)	0.5025 (14)	0.057 (9)*	
H4C	0.388 (2)	0.102 (8)	0.4961 (12)	0.043 (8)*	
H4D	0.420 (2)	0.315 (7)	0.4564 (10)	0.032 (6)*	

### Atomic displacement parameters (Å^2^)

**Table:**

	*U*^11^	*U*^22^	*U*^33^	*U*^12^	*U*^13^	*U*^23^
O11	0.0230 (6)	0.0326 (7)	0.0115 (5)	−0.0014 (5)	−0.0003 (4)	0.0001 (5)
O21	0.0174 (6)	0.0355 (7)	0.0184 (6)	0.0022 (5)	−0.0026 (4)	−0.0009 (5)
N11	0.0193 (7)	0.0201 (7)	0.0169 (7)	0.0002 (5)	0.0015 (5)	0.0005 (5)
N21	0.0234 (7)	0.0226 (7)	0.0140 (6)	−0.0029 (6)	−0.0012 (6)	0.0007 (5)
N31	0.0204 (7)	0.0308 (8)	0.0160 (7)	0.0036 (6)	−0.0001 (5)	−0.0042 (6)
C11	0.0186 (8)	0.0222 (8)	0.0225 (8)	0.0031 (6)	−0.0017 (6)	0.0005 (6)
C21	0.0224 (8)	0.0214 (8)	0.0189 (8)	−0.0019 (6)	−0.0049 (6)	0.0019 (7)
C31	0.0190 (8)	0.0168 (7)	0.0133 (7)	−0.0055 (6)	−0.0001 (5)	0.0000 (6)
C41	0.0158 (7)	0.0179 (7)	0.0131 (7)	−0.0029 (6)	0.0006 (6)	0.0006 (5)
C51	0.0178 (8)	0.0201 (7)	0.0145 (7)	−0.0060 (6)	−0.0006 (5)	0.0014 (6)
O12	0.0280 (6)	0.0322 (7)	0.0133 (5)	0.0041 (5)	0.0000 (5)	−0.0021 (5)
O22	0.0226 (6)	0.0423 (8)	0.0193 (6)	0.0091 (6)	0.0021 (5)	−0.0012 (6)
N12	0.0183 (6)	0.0220 (7)	0.0162 (6)	−0.0019 (5)	−0.0004 (5)	−0.0018 (5)
N22	0.0234 (7)	0.0237 (7)	0.0151 (6)	−0.0040 (6)	0.0019 (5)	−0.0016 (5)
N32	0.0227 (7)	0.0325 (8)	0.0144 (7)	0.0024 (6)	−0.0005 (6)	0.0043 (6)
C12	0.0190 (8)	0.0224 (9)	0.0220 (8)	0.0008 (6)	0.0014 (6)	−0.0026 (7)
C22	0.0228 (8)	0.0217 (8)	0.0203 (8)	−0.0029 (6)	0.0055 (6)	−0.0050 (6)
C32	0.0187 (8)	0.0192 (7)	0.0146 (7)	−0.0054 (6)	−0.0004 (6)	−0.0004 (6)
C42	0.0176 (7)	0.0187 (8)	0.0126 (7)	−0.0032 (6)	0.0006 (6)	−0.0012 (6)
C52	0.0198 (8)	0.0211 (8)	0.0158 (7)	−0.0014 (6)	0.0025 (6)	−0.0017 (6)
N3	0.0196 (7)	0.0230 (7)	0.0178 (7)	−0.0004 (6)	−0.0007 (5)	−0.0002 (6)
N4	0.0227 (7)	0.0348 (9)	0.0174 (7)	−0.0044 (6)	−0.0042 (6)	0.0037 (7)

### Geometric parameters (Å, °)

**Table:**

O11—C51	1.266 (2)	N22—C22	1.337 (2)
O21—C51	1.250 (2)	N22—C32	1.353 (2)
N11—C41	1.329 (2)	N32—C32	1.346 (2)
N11—C11	1.339 (2)	N32—H32A	0.88 (3)
N21—C21	1.333 (2)	N32—H32B	0.86 (3)
N21—C31	1.351 (2)	C12—C22	1.386 (3)
N31—C31	1.341 (2)	C12—H12	0.9500
N31—H31A	0.92 (3)	C22—H22	0.9500
N31—H31B	0.90 (3)	C32—C42	1.440 (2)
C11—C21	1.383 (2)	C42—C52	1.517 (2)
C11—H11	0.9500	N3—H3A	0.92 (3)
C21—H21	0.9500	N3—H3B	0.93 (3)
C31—C41	1.434 (2)	N3—H3C	0.96 (3)
C41—C51	1.518 (2)	N3—H3D	0.89 (3)
O12—C52	1.256 (2)	N4—H4A	0.86 (3)
O22—C52	1.260 (2)	N4—H4B	0.95 (4)
N12—C42	1.320 (2)	N4—H4C	0.84 (3)
N12—C12	1.340 (2)	N4—H4D	0.87 (3)
			
C41—N11—C11	119.14 (15)	N12—C12—H12	119.8
C21—N21—C31	117.49 (15)	C22—C12—H12	119.8
C31—N31—H31A	118.4 (17)	N22—C22—C12	122.70 (16)
C31—N31—H31B	119.6 (17)	N22—C22—H22	118.7
H31A—N31—H31B	121 (2)	C12—C22—H22	118.7
N11—C11—C21	120.16 (16)	N32—C32—N22	117.48 (15)
N11—C11—H11	119.9	N32—C32—C42	122.70 (16)
C21—C11—H11	119.9	N22—C32—C42	119.81 (15)
N21—C21—C11	122.88 (16)	N12—C42—C32	120.68 (15)
N21—C21—H21	118.6	N12—C42—C52	116.10 (14)
C11—C21—H21	118.6	C32—C42—C52	123.17 (15)
N31—C31—N21	117.27 (15)	O12—C52—O22	125.71 (15)
N31—C31—C41	122.85 (15)	O12—C52—C42	116.79 (14)
N21—C31—C41	119.88 (15)	O22—C52—C42	117.50 (15)
N11—C41—C31	120.42 (15)	H3A—N3—H3B	107 (2)
N11—C41—C51	115.96 (14)	H3A—N3—H3C	111 (2)
C31—C41—C51	123.62 (14)	H3B—N3—H3C	116 (2)
O21—C51—O11	125.19 (15)	H3A—N3—H3D	111 (2)
O21—C51—C41	118.80 (14)	H3B—N3—H3D	108 (2)
O11—C51—C41	116.01 (14)	H3C—N3—H3D	103 (2)
C42—N12—C12	119.08 (15)	H4A—N4—H4B	119 (3)
C22—N22—C32	117.32 (15)	H4A—N4—H4C	113 (3)
C32—N32—H32A	119.4 (16)	H4B—N4—H4C	97 (3)
C32—N32—H32B	119.9 (18)	H4A—N4—H4D	106 (3)
H32A—N32—H32B	116 (2)	H4B—N4—H4D	107 (3)
N12—C12—C22	120.40 (16)	H4C—N4—H4D	114 (3)
			
C41—N11—C11—C21	1.6 (3)	C42—N12—C12—C22	0.1 (2)
C31—N21—C21—C11	−0.2 (3)	C32—N22—C22—C12	0.2 (3)
N11—C11—C21—N21	−1.4 (3)	N12—C12—C22—N22	−0.5 (3)
C21—N21—C31—N31	−178.70 (15)	C22—N22—C32—N32	−179.47 (15)
C21—N21—C31—C41	1.5 (2)	C22—N22—C32—C42	0.5 (2)
C11—N11—C41—C31	−0.2 (2)	C12—N12—C42—C32	0.5 (2)
C11—N11—C41—C51	−179.62 (15)	C12—N12—C42—C52	−176.88 (15)
N31—C31—C41—N11	178.87 (16)	N32—C32—C42—N12	179.09 (15)
N21—C31—C41—N11	−1.3 (2)	N22—C32—C42—N12	−0.8 (2)
N31—C31—C41—C51	−1.8 (2)	N32—C32—C42—C52	−3.7 (2)
N21—C31—C41—C51	178.00 (15)	N22—C32—C42—C52	176.38 (14)
N11—C41—C51—O21	177.16 (15)	N12—C42—C52—O12	4.3 (2)
C31—C41—C51—O21	−2.2 (2)	C32—C42—C52—O12	−173.05 (16)
N11—C41—C51—O11	−3.6 (2)	N12—C42—C52—O22	−176.49 (15)
C31—C41—C51—O11	177.04 (15)	C32—C42—C52—O22	6.2 (2)

### Hydrogen-bond geometry (Å, °)

**Table:**

*D*—H···*A*	*D*—H	H···*A*	*D*···*A*	*D*—H···*A*
N31—H31A···N22^i^	0.92 (3)	2.20 (3)	3.103 (2)	169 (2)
N31—H31B···O21	0.90 (3)	2.07 (3)	2.726 (2)	129 (2)
N32—H32A···N21^ii^	0.88 (3)	2.23 (3)	3.100 (2)	168 (2)
N32—H32B···O22	0.86 (3)	2.06 (3)	2.686 (2)	129 (2)
N3—H3B···O21	0.93 (3)	1.97 (3)	2.849 (2)	157 (2)
N3—H3C···O11^iii^	0.96 (3)	2.58 (3)	3.287 (2)	131 (2)
N3—H3C···N11^iii^	0.96 (3)	2.00 (3)	2.909 (2)	159 (2)
N3—H3D···O11^iv^	0.89 (3)	2.13 (3)	2.944 (2)	152 (2)
N4—H4A···O12	0.86 (3)	2.13 (3)	2.897 (2)	148 (3)
N4—H4A···N12	0.86 (3)	2.23 (3)	2.912 (2)	135 (3)
N4—H4B···O11	0.95 (4)	1.86 (4)	2.793 (2)	166 (3)
N4—H4C···O11^v^	0.84 (3)	2.01 (4)	2.839 (2)	170 (3)
N4—H4D···O22^vi^	0.87 (3)	1.87 (3)	2.742 (2)	176 (3)

Symmetry codes: (i) −*x*+1/2, *y*+1, *z*+1/2; (ii) −*x*+1/2, *y*, *z*−1/2; (iii) *x*−1/2, −*y*+1, *z*; (iv) *x*−1/2, −*y*+2, *z*; (v) *x*, *y*−1, *z*; (vi) *x*+1/2, −*y*+1, *z*.

## References

[bb1] Abrahams, S. C. & Keve, E. T. (1971). *Acta Cryst.* A**27**, 157–165.

[bb2] Bruker (2010). *APEX2* and *SAINT* Bruker AXS Inc., Madison, Wisconsin, USA.

[bb3] Dobson, A. J. & Gerkin, R. E. (1996). *Acta Cryst.* C**52**, 1512–1514. 10.1107/s0108270195016416 8766897

[bb4] Ellsworth, J. M. & zur Loye, H.-C. (2008). *Dalton Trans.* pp. 5823–5835. 10.1039/b807227b 19082035

[bb5] Mackay, A. L. (1984). *Acta Cryst.* A**40**, 165–166.

[bb6] Macrae, C. F., Edgington, P. R., McCabe, P., Pidcock, E., Shields, G. P., Taylor, R., Towler, M. & van de Streek, J. (2006). *J. Appl. Cryst.* **39**, 453–457.

[bb7] Ptasiewicz-Bak, H. & Leciejewicz, J. (1997). *Pol. J. Chem.* **71**, 1350–1358.

[bb8] Ptasiewicz-Bak, H. & Leciejewicz, J. (1999). *Pol. J. Chem.* **73**, 717–725.

[bb9] Ptasiewicz-Bak, H., Leciejewicz, J. & Zachara, J. (1995). *J. Coord. Chem.* **36**, 317–326.

[bb10] Schomaker, V. & Trueblood, K. N. (1998). *Acta Cryst.* B**54**, 507–514.

[bb11] Sheldrick, G. M. (2008*a*). *SADABS* University of Göttingen, Germany.

[bb12] Sheldrick, G. M. (2008*b*). *Acta Cryst.* A**64**, 112–122. 10.1107/S0108767307043930 18156677

[bb13] Spek, A. L. (2009). *Acta Cryst.* D**65**, 148–155. 10.1107/S090744490804362X PMC2631630 19171970

